# An Integrative Thrombosis Network: Visualization and Topological Analysis

**DOI:** 10.1155/2015/265303

**Published:** 2015-05-17

**Authors:** Xiangjun Kong, Wenxia Zhou, Jian-Bo Wan, Qianru Zhang, Jingyun Ni, Yuanjia Hu

**Affiliations:** ^1^State Key Laboratory of Quality Research in Chinese Medicine, Institute of Chinese Medical Sciences, University of Macau, Macau; ^2^Institute of Pharmacology and Toxicology, Academy of Military Medical Sciences, Beijing 100850, China; ^3^Pharmacy School, Zunyi Medical College, Zunyi, Guizhou 563000, China

## Abstract

A comprehensive understanding of the integrative nature of the molecular network in thrombosis would be very helpful to develop multicomponent and multitarget antithrombosis drugs for use in traditional Chinese medicine (TCM). This paper attempts to comprehensively map the molecular network in thrombosis by combining platelet signaling, the coagulation cascade, and natural clot dissolution systems and to analyze the topological characteristics of the network, including the centralities of nodes, network modules, and network robustness. The results in this research advance understanding of functions of proteins in the thrombosis network and provide a reference for predicting potential therapeutic antithrombotic targets and evaluating their influence on the network.

## 1. Introduction

Thromboembolic disorders are a major cause of death and disability and affect millions worldwide. Thrombosis can occur in either the arterial or the venous circulation and results in different clinical symptoms, such as pulmonary emboli, deep vein thrombosis, strokes, and heart attacks.

While antithrombotic drugs, including anticoagulants, antiplatelet drugs, and thrombolytic drugs, have been widely used for the prevention and treatment of arterial and venous thrombosis, new targets, more effective agents against existing targets, as well as new therapeutic strategies still need to be developed for overcoming resistance to current drugs, suppressing the stimulus in platelet activation, and regulating the anticoagulation effect more conveniently [1].

Traditional Chinese medicine (TCM), especially HuoXueHuaYu Chinese medicines, has long been used to treat thrombosis. The significant efficacy of TCM in treating thrombosis has been reported in the literature and in recent pharmacological experiments [2–4]. Thus, TCM seems to offer a possible route to the discovery of new targets, agents, and therapeutic strategies for the treatment of thrombosis. It is widely believed that the mechanism of multicomponent and multitarget may be of great essence for TCM to exert integrative treatment effects [5–7]. To better understand the potential of TCM in the treatment of thrombosis, the molecular network involved in the disease needs to be elucidated.

Moreover, several studies have been so far conducted to evaluate the efficacy of different compounds against platelet aggregation, exhibit formula-target relations, and develop model to predict coagulation response [8–10]. However, little is known about the system-wide effects of molecules of thrombosis from a holistic perspective with the comprehensive consideration between efficacy and safety referring to the balance of antithrombosis and bleeding.

On the other hand, the rapid progress of bioinformatics and systems biology has provided not only a systems-level understanding of biological processes and disease complexity but also an efficient and promising approach, such as network analysis, for integrative drug development [5, 11]. Csermely et al. presented a comprehensive review of analytical tools of network topology and dynamics and advances in applications for drug discovery [12]. Moreover, potential targets were identified by detecting key nodes in a disease-specific network with important topological properties [13, 14].

In this context, this research attempts to comprehensively map the thrombosis molecular network and analyze topological characteristics of the network from several perspectives, including the centralities of nodes, network modules, and network robustness. This research is of significance to improve the understanding of molecular functions in the thrombosis network and further predict potential targets for the treatment of thrombosis by evaluating their influence on the network.

## 2. Methods

### 2.1. Network Construction

**Figure 1:**
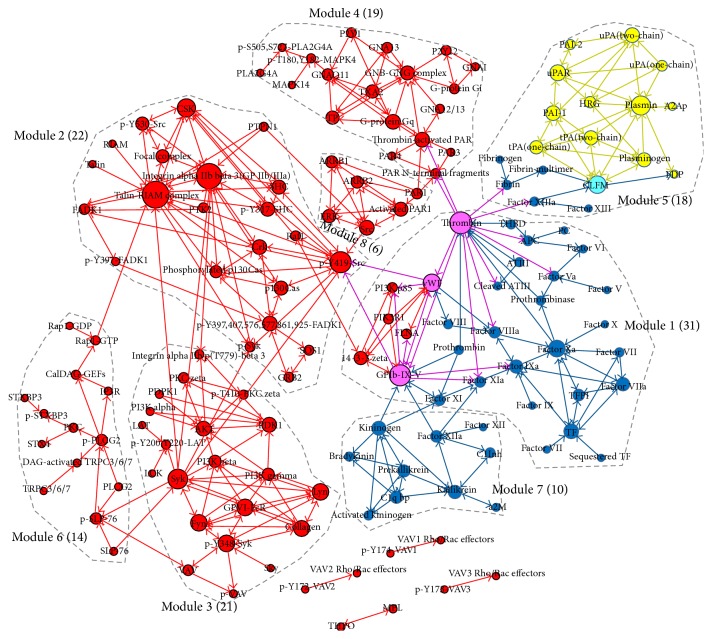
Visualized thrombosis network. The colors of the different nodes represent their involvement in diverse functions in thrombosis, as determined from the Reactome pathway analysis. The red node means protein taking part in platelet activation, signaling, or aggregation, which owns most participants. The blue and yellow represent function of clotting cascade and fibrin clot dissolution process, respectively. The size of node corresponds to its degree. Nodes involved in a module are marked within the largest component, and modules are sorted by number of involved nodes.

Reactome is a curated and peer-reviewed pathway database that functions as a data-mining resource and electronic textbook, with the focus on* Homo sapiens* [15]. Details of pathways, such as constituent reactions and participating complexes and relationships, are elucidated in Reactome. We retrieved pathways and reaction information from the Reactome database that were relevant to thrombosis. These included (1) platelet activation, signaling, and aggregation, (2) the clotting cascade, and (3) the dissolution of fibrin clots. We organized these pathways and reactions as elementary reactions that contained one reactant and its corresponding product, regardless of small molecules. In this step, protein complexes were involved in the majority of elementary reactions. To identify potentially effective therapeutic targets against thrombus, we split the complexes in the elementary reactions into separate single proteins forming reactant group and product group. Then, the splitting proteins are reconnected from each reactant to each different product, except self-connections. Finally, the relations between the proteins with reacting directions were detected. Gephi software (http://gephi.github.io/) was then used to construct an evidence-based and integrative thrombosis network (Figure 1) [16].

In this network, nodes represent proteins related to thrombosis, and edges with direction between nodes indicate their interacting connections. The direction of the edges denotes the reaction stream, from the node at the start of the arrow to the node downstream at the end of the arrow. The edge of a double-headed arrow denotes the bidirectional reaction of a protein pair. Based on the principle of network generation, the double-headed arrows imply that the proteins function in complexes.

### 2.2. Centrality Analysis

The centrality definition of a node in a network is related to the concept of importance. Dozens of centrality measures have been developed to understand network structure, and these have been widely used to find central nodes in various biological systems [12, 17]. In this research, we examined the node degree, betweenness centrality, and closeness centrality of the nodes to shed light on key druggable proteins that might serve as targets in thrombosis. The centralities are calculated based on the algorithms referred by Gephi [18].

The degree of node *v*, *C*_deg⁡_(*v*), is calculated by the following equation:

(1) $$\begin{array}{c} C_{\deg}\left(v\right)=\underset{u\in V,u\neq v}{\sum }d\left(v,u\right), \end{array}$$

where *d*(*v*, *u*) is 1, if and only if node *v* and node *u* are connected by an arrow, no matter where *v* positions are (the start or the end of the arrow); otherwise, it is 0. Nodes *u* and *v* are different nodes from node set *V* of network. Thus, degree is limited in the scope of nodes that are directly connected to a node, but not including the indirect connections.

**Figure 2:**
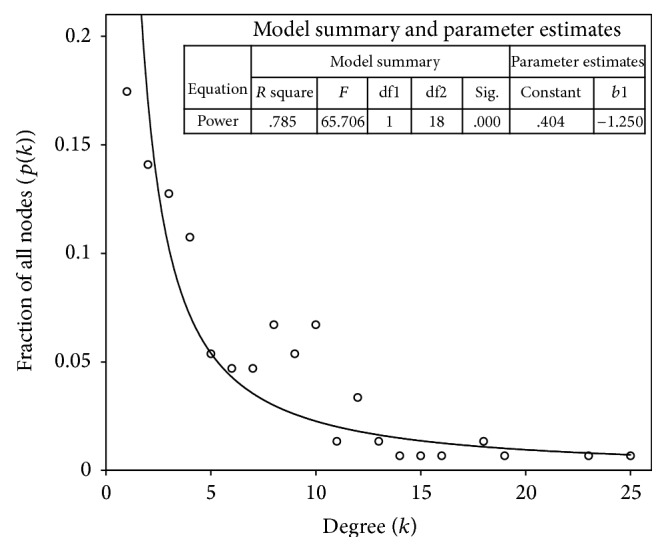
The degree distribution of the thrombosis network (scatter spot and fitting curve in power law). The horizontal axis denotes the number of connections of the nodes and the vertical denotes the fraction of nodes with a specific degree. The distribution fitted a power law, with *γ* = 1.25, *p* < 0.001.

We used *k* to denote a node's degree in thrombosis network. Then, we tested the degree distribution *p*(*k*) of the network, giving the fraction of nodes with degree *k*, (*k* = 1,2,…) (Figure 2). We performed a goodness-of-fit test to determine the degree distribution of the constructed thrombosis network whether it follows power-low. The hub of a network refers to a node with a much higher degree than the average. The network hubs are listed by degree order from high to low in Table 1.

The betweenness centrality of node *v*, *C*_bet_(*v*), measures the number of shortest paths that pass through the node:

(2) $$\begin{array}{c} C_{\text{bet}}\left(v\right)=\underset{u,w\in V,u\neq v\neq w}{\sum }\frac{\sigma_{u,w}\left(v\right)}{\sigma_{u,w}}, \end{array}$$

where *σ*_*u*,*w*_(*v*) is the number of shortest paths from node *u* to node *w* that pass through the node *v*; *σ*_*u*,*w*_ is the number of shortest paths between node *u* and node *w*. A node with high betweenness centrality serves as a bridge between other nodes in the whole network. Thus, the communication between other nodes becomes more dependent on this node in the network.

Unlike the degree, closeness centrality of a node examines the direct and indirect links connected to the nodes. The closeness centrality of node *v*, *C*_clo_(*v*), is the mean shortest path of the node connecting to all other nodes in the network:

(3) $$\begin{array}{c} C_{\text{clo}}\left(v\right)=\frac{\sum_{u\in V,u\neq v}\text{dis}\left(v,u\right)}{\sum_{u\in V,u\neq v}n\left(v,u\right)}, \end{array}$$

where dis(*v*, *u*) denotes the distance between nodes *v* and *u*, that is, the minimum length of any path connecting *v* and *u* in network. In this equation, *n*(*v*, *u*) is 1, if there is a path linking node *v* and node *u*; otherwise, it is 0. For an isolated node, its closeness centrality is 0.

### 2.3. Identification of Network Module

Network modules are classical measures of mesoscopic network structures. A group of nodes that is connected more closely to group members than others outside this group is regarded as a module or a community that has fewer connections between modules. In this paper, the module detection Louvain algorithm incorporated in Gephi was used to explore the modularity structure of the network [19]. The modular function was then analyzed to shed light on the complex relationship among the modules. The modular hubs (i.e., the nodes with a higher degree than the other nodes in the same module) are listed in Table 2.

### 2.4. Analysis of the Robustness of the Network

The robustness of a network reflects the tolerance of a network to failures or its ability to withstand attacks. Robust networks maintain the stability of system function against failures or attacks. Drug action often fails or generates serious side effects due to high network robustness or hitting unexpected points of networks [20–22]. Here, in order to identify potential drug targets, we investigated the robustness of a thrombosis network under the simulation of random failure or a deliberate attack. Random failures of cellular network are usually caused by the oxidative damage, the indirect effect from somatic mutations, and complex influence of ageing [23, 24], while deliberate attacks refer to drug-driven influence to network. As introduced by Albert et al., we used the indicator *S* and 〈*s*〉 to evaluate the network robustness and fragmentation process [25]. When a fraction, *f*, of all the network nodes was removed randomly (failure) or removed as degree order (hub attack) or betweenness centrality order (bridge attack) of nodes, we calculated the fraction of the size of the largest component comparing to the total system size, *S*. Then, we detected the average size 〈*s*〉 of the isolated components (all the components except the largest one) when the same fraction of nodes was removed. The behavior of the network, with an increasing *f*, is presented in Figure 4.

## 3. Results and Discussion

### 3.1. Visualization of the Network

We constructed a human thrombosis network by combining serial signal pathways of activating and recruiting platelets initiating blood coagulation and generating thrombi and fibrin. These events occur concomitantly (Figure 1). The resulting thrombosis network provides a visual and relatively integrative perspective to understand thrombosis in various diseases.

There are 149 proteins and 414 relations in the network, which is made up of one large component and four small separated components. The network is composed of the three parts of functions that connect with each other. It includes most of the receptors and enzymes involved in these three factors, such as integrin alpha IIb beta 3 (GP IIb/IIIa), antithrombin III (ATIII), glycoproteins of the Ib, IX, and V complex (GPIb-IX-V), von Willebrand factor (vWF), thrombin, proteinase-activated receptor 1 (PAR1), P2Y12, P2Y1, tissue pathway factor inhibitor (TFPI), plasminogen activator inhibitor-1 (PAI-1), and plasminogen activator inhibitor-2 (PAI-2). Interestingly, four nodes exhibited two diverse functions: GPIb-IX-V, vWF, and thrombin were particularly important contributors to both platelet signaling and the coagulation cascade; the cross-linked fibrin multimer (CLFM) was the common target of the coagulation and natural clot dissolution system. We consider the four proteins (GPIb-IX-V, vWF, thrombin, and CLFM) as multifunctional proteins. The average degree of each node was 2.78, and the average shortest path length was 5.37.

The degree distribution *p*(*k*) is an important measure of the topological features of the network (Figure 2) [26]. The degree distributions of most real-world networks, including biological networks, follow a power law, *p*(*k*) ~ *Ak* ^−*γ*^, where *γ* is the power-law exponent. The degree distribution in network generated in this way obeys the following power law: *p*(*k*) = *Ak* ^−1.25^, *p* < 0.001. The degree distribution of the thrombosis network was approximately scale free (when 2 < *γ* < 3). As confirmed by the power law, most of the nodes in this network only influenced a limited number of other nodes, and a small number of nodes interacted with many other nodes. These nodes are likely to play key roles in the functional system [27].

### 3.2. Identification of Key Targets

Hubs with a high degree of centrality occupy a critical position in a network, although they house only a small number of all the nodes in a network. If hubs are attacked, the integrity of the network deteriorates more rapidly than nonhubs, which makes hubs attractive drug targets [25]. Thus, it is useful to study the key proteins contributing to thrombus formation as network hubs. Therefore, hubs with degrees larger than 10 and their topological properties were extracted (Table 1).

Table 1 shows 27 hubs with diverse functions including the four multifunctional proteins. The locations of multifunctional proteins indicate the mutually influential relation among the three functions in formation of thrombi. Among these hubs, many have been well developed as effective antithrombotic targets, involving U.S. Food and Drug Administration- (FDA-) approved therapeutic targets and preclinical developing targets. Thrombin, factor Xa, GP IIb/IIIa, PAI-1, and urokinase plasminogen activator receptor (uPAR) are typical targets of popular clinical medicines [28, 29]. However, there are plenty of proteins with a high degree, such as the Rap1-interacting adaptor molecule (RIAM) complex, that are not suitable for drug development [30].

On average, the targets of FDA-approved drugs tend to have more connections than most peripheral nodes but do not cover all the hubs [31]. Hub connectors, such as factor XIa, factor IXa, and 14-3-3 zeta, that connect GPIb-IX-V, ATIII, and factor V with thrombin and connect plasminogen with CLFM are linked to major hubs and provide very interesting targeting options [24].

Different centrality measures indicate different importance of nodes in the network. Nodes with high betweenness centrality indicate their particular targeting potential for antithrombosis due to their bottleneck positions in the thrombosis network. It should be noted that 16 out of 27 hubs also have high betweenness centrality. Moreover, high degree and betweenness centralities exhibit essential topological significance in thrombosis network by serving as network hubs and bridges.

On the other hand, a substantial number of key proteins in Table 1 are enzymes related to cell survival, growth, and metabolism and activate or promote the development of thrombosis signaling series, such as Src family kinases, the PI3K/AKT pathway, and Syk. The topological positions of enzymes in network highlight their potential roles as therapeutic targets. Enzyme signal pathways are increasingly recognized as targets of antithrombosis drugs. The activation of Src family kinases (SFKs), a family containing eight structurally related tyrosine kinases, namely, Lyn, Fyn, Src, Fgr, Blk, Hck Yes, and Lck, is an important event downstream of integrin adhesion signaling that is involved in initiating and amplifying signals in platelets [32, 33]. Research on mice has provided preliminary but important implications for exploring inhibitors targeting individual SFKs, in particular, Lyn [32]. The central role of Syk identified by both high degree and betweenness centrality in numerous signaling cascades also highlights its promise in the development of novel antithrombotic therapeutics [34]. All these appear to be consistent with the prediction derived from network centrality implication exhibited in Table 1. However, as these enzymes have multiple roles in other biological processes, an appropriate drug-delivery system is needed that specifically targets the thrombus system.

Nodes with overlapping function are key determinants of network cooperation. Overlapping nodes occupy specific network positions and can provide more subtle regulation. As shown in Figure 1 and Table 1, four multifunctional proteins cross-linking coagulation cascade and platelet signaling or clot dissolution affect both sides broadly, due to their high degree and betweenness centralities. The potential of GPIb-IX-V and vWF as antiplatelet adhesion targets has been investigated in mounting evidence from basic research and clinical evaluations for antiplatelet agents identification [35, 36]. Inhibitors against thrombin are also the focus of much research to improve the treatment of thrombus [37].

**Figure 3:**
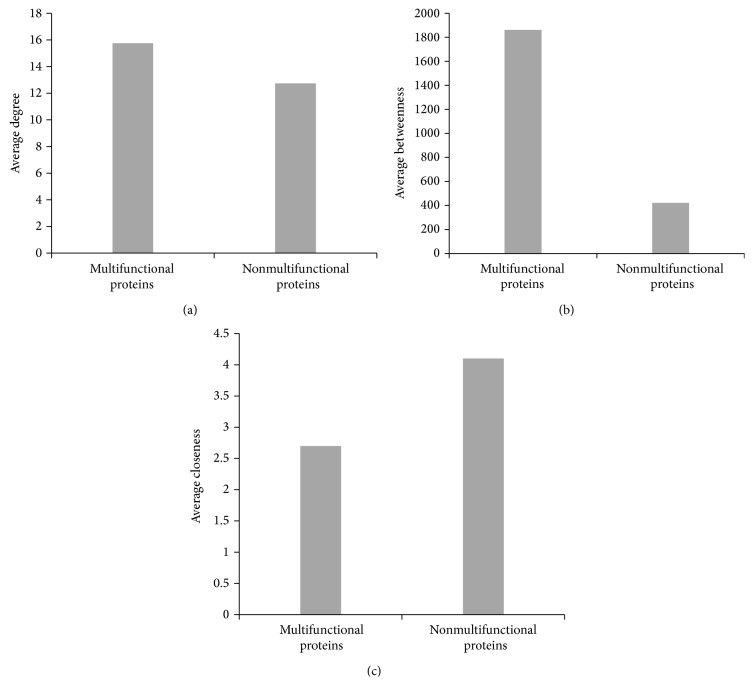
The centralities of multifunctional and nonmultifunctional proteins: (a) average degree; (b) average betweenness; (c) average closeness.

We compared the average degree, betweenness centrality, and closeness centrality of multifunctional proteins with those of the nonmultifunctional proteins shown in Table 1 to identify their specific topological characteristics.  Figure 3 shows the difference in the betweenness and closeness centrality of these multifunctional and nonmultifunctional proteins. Multifunctional nodes bridging the three components of thrombosis (i.e., platelet signaling, the coagulation cascade, and the natural clot dissolution system) show much higher betweenness and interact with other nodes closely. These likely contribute to such encouraging performance of functional overlaps as attractive targets for antithrombotic treatment.

### 3.3. Implications of Network Modularity

To facilitate the interpretation of the complex relationships in the thrombosis system, the modular structure of the system was explored. We marked eight modules positioned in the largest component in Figure 1 and sorted them by the number of involved nodes in Table 2. The mechanism of each module and the functional interdependencies among the modules are illustrated in Table 2. The findings provide insight into the complex biological process of thrombosis corresponding to the functional modules' network positions.

In module 1, GPIb-IX-V and vWF not only were important components of platelet adhesion but also strongly associated with the classic coagulation cascade by factor XI and factor VIII [38]. In addition, thrombin, as the most potent platelet agonist, coordinates the process of platelet activation and aggregation with coagulation [1]. Together with factor Xa [39], they serve as modular hubs and are considered important targets in antithrombotic treatment. Network hubs are scattered throughout diverse modules. Most are modular hubs.

Otherwise, modular hubs which are not network hubs should also be emphasized in view of their local influence on some specific functions. For example, phosphorylated phospholipase C gamma 2 (p-PLCG2), as a hub of module 6, and kininogen and prekallikrein as hubs in the kallikrein-kinin system have been demonstrated by previous studies to show the potential as antithrombotic targets [40–43]. Another study also showed that hub-related properties significantly affected modular functions, making them attractive network drug targets when partial modulating against specific thrombosis processes [44]. The aforementioned suggests that putative targets can be identified by their modular status as well.

### 3.4. Analysis of Network Robustness

Robustness is an intrinsic property of networks. It refers to the ability of a network to continue functioning in the face of various perturbations. The action of drugs can be perceived as a disease network perturbation modulating disordered network towards a functional state [45, 46]. Drugs that target a single node destroy the connections between that node and other nodes. In this context, a network approach can shed light on the effect of different drugs on various targets.

**Figure 4:**
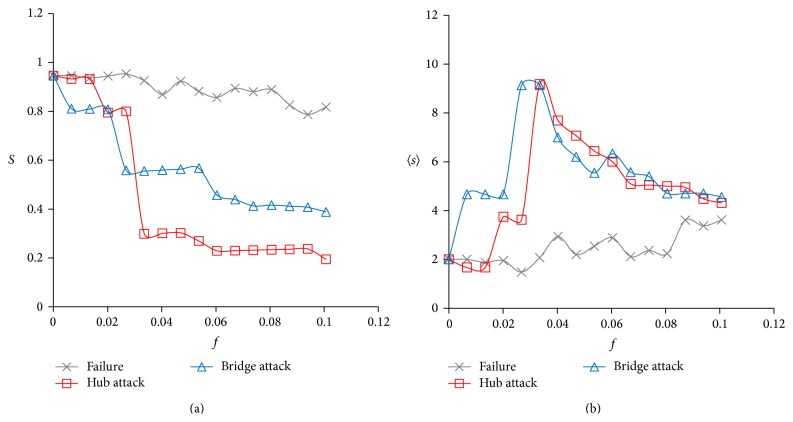
Network fragmentation under random failures, hub attacks, and bridge attacks measured by (a) the relative size of the largest cluster *S* and (b) the average size of the isolated components 〈*s*〉.

Due to advances in the theoretical understanding of network structure, it is possible to quantitatively describe a network with graph concepts. As the degree distribution of the thrombosis network conforms to power law, and the network is relatively scale free (Section 3.1), it is likely resistant to random damage but sensitive to the targeted removal of nodes [25]. Networks have a number of vulnerable points, such as hubs and bridges, and they can be attacked at any of these. In this paper, we simulated hub attacks and bridge attacks to examine the robustness of a thrombosis network (Figure 4).

As shown in Figure 4, the response of the thrombosis network to attacks and failures differed. When nodes were removed continuously from the network (up to *f* = 0.1), the size of the largest component *S* remained the dominating position under random failure but fell apart to moderate size obviously when hubs or bridges were attacked (Figure 4(a)). When one or two nodes were removed, only bridge attacks had much of an effect on the network, pointing to the importance of targeting nodes with high betweenness when developing single-target agents. As *f* increased, the size of the network largest component decreased more rapidly under hub attacks than bridge attacks. When hubs were attacked, *S* displayed threshold-like behavior. At *f* ≈ 0.03 (about five nodes were removed), *S* ≈ 0.2, and the network experienced catastrophic fragmentation. As shown in an earlier study, the fragmentation would break off continuously but less severely when larger fraction than 0.1 of nodes was removed [25].

The fact that the average size 〈*s*〉 of the isolated components increased slowly indicated that increasing failure level led to the isolation of single nodes, not large components (Figure 4(b)). In the attack mode, the system was sensitive to the removal of key nodes and was separated into certain size of components, which explains the rapid increased 〈*s*〉 for the small *f*. Similar threshold of 〈*s*〉 was detected in attack mode, where the main component broke into small pieces and also led to the size of fragments peaks. As we continued to remove nodes, the isolated components became deflated, leading to a descending 〈*s*〉. The aforementioned behavior provides evidence that the thrombosis network shows topological stability against random failures but that it fragments in response to attacks on a small number of nodes. Obviously, bridge attacks are more sensitive than attacks on hubs, and hub attacks cause more serious fragmentation of the network. These observations of the global influence of network attacks could provide clues for seeking fragile targets and designing multitarget therapeutic strategies against thrombosis.

## 4. Conclusions

Network analysis has the advantage of providing system-level perspectives on complex issues. Topological analysis can help to extract valuable information hiding in large-scale and complex experimental data. In summary, on the foundation of evidence-based data, we constructed an integrated thrombosis network composed of platelet signaling, the coagulation cascade, and the natural clot dissolution system and conducted various network topological analyses. The degree distribution followed a power law, and the network was relatively scale free. With this in mind, local topology analysis was conducted to identify central nodes that could be putative drug targets. The results showed that targets can also be predicted from their modular position by modularity analysis. The analysis of the robustness of the thrombosis network demonstrated that it was highly resistant to random failure but sensitive to hub and bridge attacks. Such studies can elucidate the function of proteins in thrombosis network, help discover new targets for the treatment of thrombus using TCM, and contribute to the development of new targets of TCM and multitarget strategies.

Network analysis seems to provide a valuable prediction of therapeutic targets, but it is still insufficient to validate the effectiveness of targets. Further pharmaceutical experiments are necessary for eventual validation of network results. Network approach can serve as a valuable complement to the experimental efforts, while a combination between simulated and experimental studies is of great significance for effective drug discovery in future.

## Figures and Tables

**Table 1:** Topological information on key proteins in the thrombosis network, ranked by degree.

Rank	Nodes	Degree	Betweenness	Closeness	Functions^a^
1	Talin-RIAM complex	25	689	6.2	Platelet signaling
2	GP IIb/IIIa	23	215	6.2	Platelet signaling
3	Thrombin^b^	19	3089	2.7	Multifunction
4	GPIb-IX-V^b^	18	2818	2.9	Multifunction
5	p-Y419-Src	18	2866	4.8	Platelet signaling
6	Syk	16	686	4.7	Platelet signaling
7	Plasmin	15	294	1.5	Clot dissolution
8	CSK	14	151	6.3	Platelet signaling
9	vWF^b^	13	542	3.4	Multifunction
10	CLFM^b^	13	992	1.7	Multifunction
11	Fyn	12	133	5.6	Platelet signaling
12	GPVI-FcR	12	133	5.6	Platelet signaling
13	Lyn	12	133	5.6	Platelet signaling
14	p-Y348-Syk	12	288	6.4	Platelet signaling
15	Factor Xa	12	1032	3.5	Coagulation
16	Collagen	11	9	6.4	Platelet signaling
17	uPAR	11	145	1.5	Clot dissolution
18	Src	10	396	5.4	Platelet signaling
19	PDK1	10	21	1.0	Platelet signaling
20	AKT	10	21	1.0	Platelet signaling
21	PAI-1	10	177	1.7	Clot dissolution
22	Crk	10	502	5.5	Platelet signaling
23	p-Y397, 407, 576, 577, 861, 925-FADK1	10	414	6.4	Platelet signaling
24	TF	10	213	4.4	Coagulation
25	Thrombin-activated PAR	10	803	1.8	Platelet signaling
26	G-protein Gq	10	338	1.5	Platelet signaling
27	uPA (two-chain)	10	53	1.6	Clot dissolution

^a^Functions refer to platelet signaling, coagulation, clot dissolution, or multifunction identification summarized from Reactome.

^b^Thrombin, GPIb-IX-V, vWF, and CLFM are multifunctional proteins serving as two functions. The first three combining functions are in platelet signaling pathways and coagulation cascades, while CLFM is in coagulation and clot dissolution system.

**Table 2:** Modularity of the thrombosis network and the identification of modular hubs.

Modules	Number of nodes	Modular hubs	Mechanisms
1	31	Thrombin, GPIb-IX-V, vWF, factor Xa	Platelet adhesion signaling and classic coagulation cascade system
2	22	Talin-RIAM complex, GP IIb/IIIa, p-Y419-Src, CSK	Platelet activation and aggregation through GP IIb/IIIa
3	21	Syk, Fyn, GPVI-FcR, Lyn, p-Y348-Syk	Platelet activation through GPVI-FcR and Syk signal
4	19	Thrombin-activated PAR, G-protein Gq, GNB-GNG complex	Accumulation of soluble agonists for platelet recruitment
5	18	Plasmin, CLFM, uPAR, PAI-1	Fibrin formation and dissolution events
6	14	p-PLCG2, p-SLP-76	Signalosome formation for promoting full platelet activation through PLCG2
7	10	Kininogen, kallikrein, C1q bp, prekallikrein	Kallikrein-kinin system
8	6	Src, activated PAR1	Typical platelet activation signal via ERK
